# Supplementary material for: The interactome of intact mitochondria by cross-linking mass spectrometry provides evidence for coexisting respiratory supercomplexes
Source: Mol Cell Proteomics. 2017 Dec 8;17(2):216–32. doi: 10.1074/mcp.RA117.000470 (PMC5795388; doi:10.1074/mcp.RA117.000470)
Supplement: Supplemental Data [file supp_RA117.000470_133922_0_supp_23978_fzffwf.zip › spectra_annotation/mito_spectra_annotation/283-1-12-1-6-1.pdf]

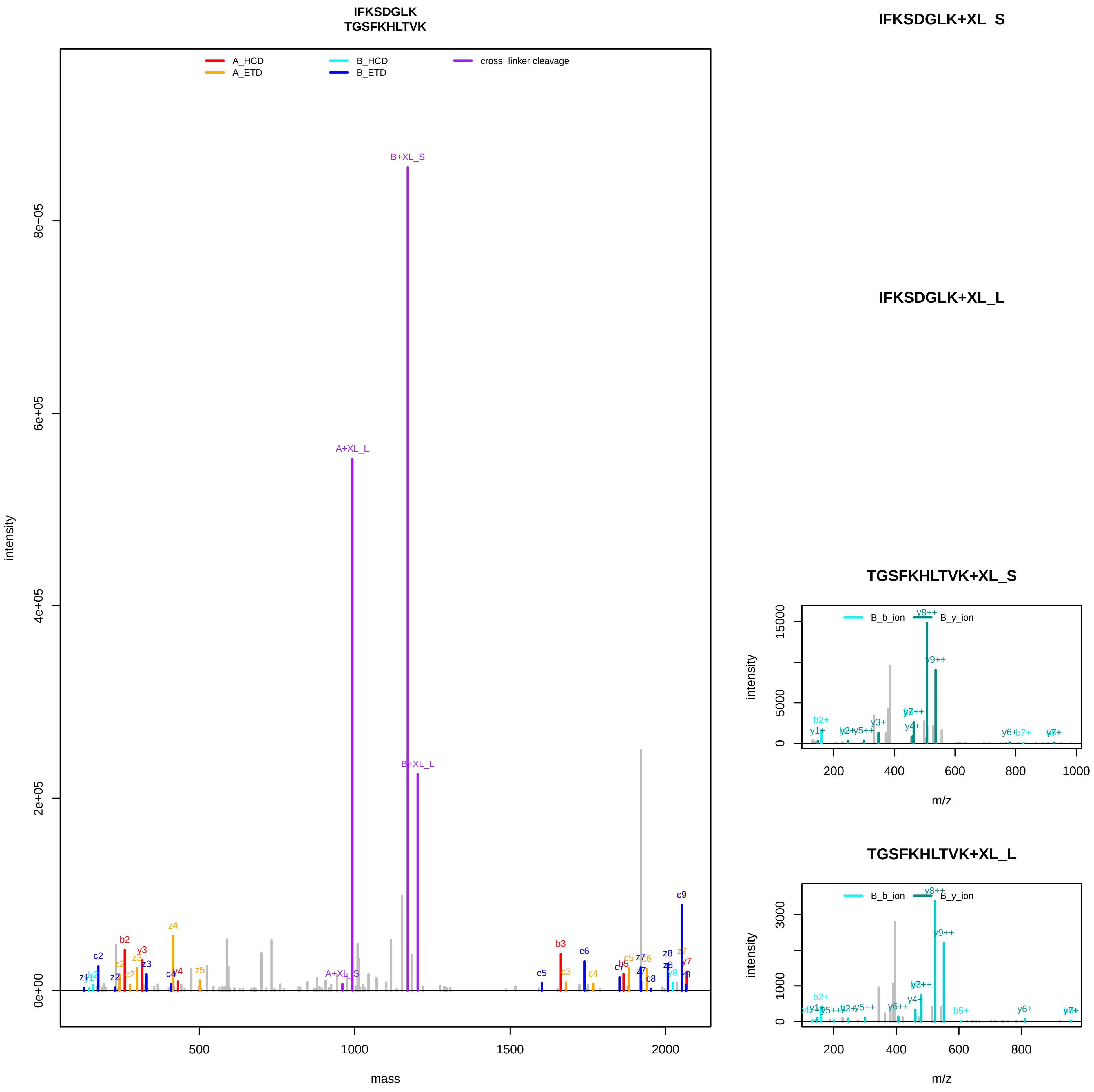

IFKSDGLK
TGSFKHLTVK
A_HCD
A_ETD
B_HCD
B_ETD
cross−linker cleavage
B+XL_S
A+XL_L
B+XL_L
A+XL_S
intensity
0e+00
2e+05
4e+05
6e+05
8e+05
500
1000
1500
2000
mass
IFKSDGLK+XL_S
IFKSDGLK+XL_L
TGSFKHLTVK+XL_S
B_b_ion
B_y_ion
y8++
y9++
intensity
0
5000
15000
200
400
600
800
1000
m/z
TGSFKHLTVK+XL_L
B_b_ion
B_y_ion
y8++
y9++
intensity
0
1000
3000
200
400
600
800
m/z
